# Mental Health Impact of the Confinement Measures During the COVID-19 Pandemic

**DOI:** 10.1093/geroni/igaa057.3484

**Published:** 2020-12-16

**Authors:** Claire Pauly, Valerie Schröder, Laure Pauly, Rejko Krüger, Anja Leist

**Affiliations:** 1 University of Luxembourg, Esch-sur-Alzette, Luxembourg; 2 Luxembourg Centre for Systems Biomedicine, Belvaux, Luxembourg; 3 Clinical and Experimental Neuroscience, Luxembourg Centre for Systems Biomedicine, University of Luxembourg, Esch-Belval, Luxembourg, Esch-sur-Alzette Belval, Luxembourg; 4 Luxembourg Institute of Health, Strassen, Luxembourg

## Abstract

Background. Mid-March 2020, with exponentially increasing COVID-19 infections, Luxembourg closed schools and businesses, and declared a crisis (état de crise) to implement confinement measures, including orders to not leave the home unless to fulfill essential needs. The psychological consequences of these policy responses to the pandemic on older people, considered a high-risk group, were unknown at the time. The aim of this study was to use the nationally representative CON-VINCE study that assessed mental health at the height of the confinement measures mid-April 2020, to assess the psychological impact of quarantine on older adults. Method. A total of 451 participants aged 60+ years (55.0% male) filled in the CES-D, the GAD-7 and the 3-item loneliness scale that measured depressive symptoms, level of anxiety, and feelings of social isolation. Results. Mean age was 67.7 years (SD 5.5), average number of school years were 13.1 (SD 3.6). The participants were mainly of Luxembourgish nationality (87.8%), and a majority (69.8%) was married. Clinically relevant depression scores were present in 13.1%, generalized anxiety in 1.8%, and self-perceived isolation in 16.9% of participants. Number of depressive symptoms was associated with perceived isolation (p<0.001) and current exercise levels (p=0.02). Discussion. The rate of older adults with clinically relevant impaired mental health was similar to pre-pandemic levels in Luxembourg, suggesting that negative mental health consequences of the confinement measures were largely absent. Possible explanations are that confinement was considered a universal experience, and that the healthcare system remained functional, unlike in other countries at the time.

